# Supplementary material for: Towards a GDPR-compliant cloud architecture with data privacy controlled through sticky policies
Source: PeerJ Comput Sci. 2024 Mar 29;10:e1898. doi: 10.7717/peerj-cs.1898 (PMC11041943; doi:10.7717/peerj-cs.1898)
Supplement: Supplemental Information 1 [file peerj-cs-10-1898-s001.pdf]

A COMPLETE ARCHITECTURAL MODEL

Figure A.1 shows, the *DataCenterElement* data type is included to represent a set of data centers with the same configuration. Likewise, the *RackElement* for racks. The profile definition includes the attributes necessary for the *component* stereotypes to simulate different system component specifications, such as the number of cores in a CPU or machines per board in a rack (*machinesPerBoard*). As can be seen, each DataCenter is composed of a set of *RackElements*, which contains a set of racks. Each *rack* component is defined by specifying the machines per board, the network, and the boards (see *Rack* component). The rack can be dedicated to computing or storage, so two types of racks are defined, namely *ComputingRack* and *StorageRack*, which contain stateless computation machines (*StalessComputationMachine* stereotype) or stateless storage machines (*SSMProcessor* stereotype), respectively. Each machine is defined in terms of CPU (*CPU* stereotype), memory (*Memory*), and storage (*Storage*). As can be seen in the bottom right of Fig. A.1, the data is associated with the *Storage* stereotype which is an attribute of the machines where it will be stored. Then, it is associated with storage and computation machines.

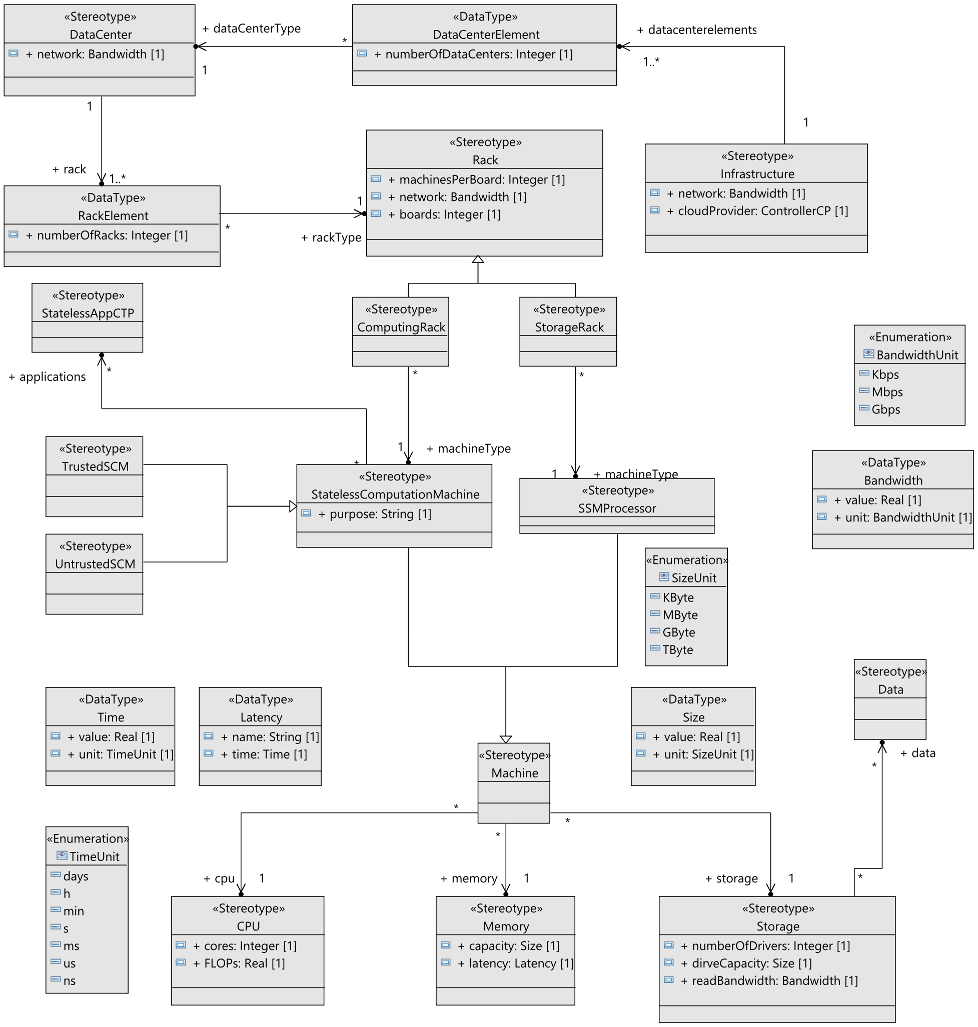

Figure A.1. Model4_DataCTrack profile: Associations and Properties of cloud-GDPR infrastructure stereotypes.

It has been necessary to define some new data and specific enumeration types. The data types created are *Time* and *Latency* (see the left part of Figure A.1), and *Size* and *Bandwidth* (right part). *Time, Size*, and *Bandwidth* consist of a value and a unit belonging to the *TimeUnit* enumerations, indicating that this time can be measured in days, hours (h), minutes (min), seconds (s), milliseconds (ms), microseconds (μs), or nanoseconds (ns) (left part of the figure). *SizeUnit* can be measured in Kilobytes, Megabytes,

Gigabytes, or Terabytes (right part). *Latency* requires a name of type string and an attribute of type *Time*. Finally, the remaining attributes consist of primitive data types, mainly integer and string, except for the *cloudProvider* attribute of the *Infrastructure* stereotype of type *ControllerCP* defined for the interaction. All these must be parameterized when defining the model.

Figure A.2 shows the attributes and the relationships between the interaction stereotypes as associations of stereotypes. Other than the relationships between *User* and *Data*, *ControllerCP* and *Data*, and *SSMProcessor* and *StatelessAppCTP*, which are regular binary relationships, all other associations model the ownership of the (opposite) end of the association. This association means that the stereotype connected by the dotted arrow will become an attribute of the stereotype associated with it (the former is owned by the latter). Therefore, most attributes are specified by another stereotype or user-defined data types, as illustrated by the *StickyPolicy* stereotype. This stereotype is made up of the following attributes: *permission*, *owners*, *purpose*, *controller*, and *accesshistory*. The *permission* attribute is required for defining restrictions (permissions) on data usage. This attribute is of the *PermissionPerTP* data type, which is used to define who is authorized to grant permissions for data access (*S*), and who has obtained permission for writing the data (*I*), both being defined as a list of lists of *tps or Users*. For this purpose, the *Principal* stereotype, which can be a *User* or a *tp*, is defined (see Section 5.2). Then, to create the list of lists, it is necessary to create a data type that establishes the first list of principals, i.e., *PList*.Thus, we can later define, in *S* and *I*, a list of this type to achieve it. The attribute *owners*, of *PList* type, establishes the user (or users in the case of combined data sets), which are data owners of the data which pairs with this policy.

Then, the *controller* attribute, of type *ControllerCP*, indicates the data controller of the data. Note that no ad-hoc identification is required as data processors usually use segmentation techniques to separate data from different data subjects. The *purpose* attribute has been extracted from point 1c of Article 13 GDPR and contains the required information, detailing the purposes for which the controller of the data allows the treatment of its data. Finally, the *accessHistory* attribute[10] of the *AccessPerTP* data type is defined to specify all the third parties that access the data, thus allowing us to track the data and obtain information about who obtained permission for that access. The *controller* and *owners* attributes, of *ControllerCP* and *User* types, respectively, indicate the data controller and the user (or users in case of combined data sets) which are data owners.

The *AccessPerTP* stereotype is used in the SP in the *accessHistory* field to track data accesses and purpose. It has three atributes: *tp*, *actionPerformed*, and *purpose*. Note that the *purpose* attribute of the *StickyPolicy* stereotype must match its contents to model that a third party does not access the data for a purpose other than the one stated by the controller.

Another important stereotype is the *AccessLog* stereotype, which represents the log used by the controller to control where data is stored and to track data accesses. A new entry will be included in the log for each access to the data to capture this. This log has the following attributes: *tp*, *l1* (l for location), *sp*, *O* (for Owners), *action*, *newl*, and *newsp*. The *tp* attribute, of *StatelessAppCTP* type (where AppCTP stands for computing application developed by a third party), relates a data access to a third party and allows us to know who is responsible for the data access. The *l1* attibute is of *Storage* type and represents the current location of the data being accessed. This attribute allows for more complete data tracking as it links a data access to a machine. The *sp* attribute, of *StickyPolicy* type, records the initial sticky policy for the data treated to detect possible alterations between the input and output data sets. The *O* attribute of type list of *Principals* (*PList*) indicates who consents to the data access. The *action* attribute is of *ActionType* type and records the operation performed on the data, which can be a read or a write. The *newl* attribute, of *Storage* type, specifies the location where the data has been stored after the action performed on it. Finally, the last property, namely *newsp*, of type *StickyPolicy*, contains the resulting policy on the data after the action. The value of this attribute when data are combined over two sets of data is shown in Section 5.2.

The *SLA* stereotype has five attributes that are modeled on the basis of Article 28 GDPR. This stereotype represents the contract that governs data processing, which the controller and processor are required to sign, in accordance with point 3 of the above article. The attributes of this stereotype are *subjectMatter*, *processingDuration*, *recipients*, *processingNature*, *processingPurpose*, and *processingInstructions*. The first two attributes, defined as an array of *strings* and *Time* stereotype, respectively, set the theme and duration of the processing. The *recipients* attribute is defined as a list of *StatelessAppCTP* and represents

[10]Note that we have added this property to track user data, but it is generally not considered in the definition of Sticky Policy.

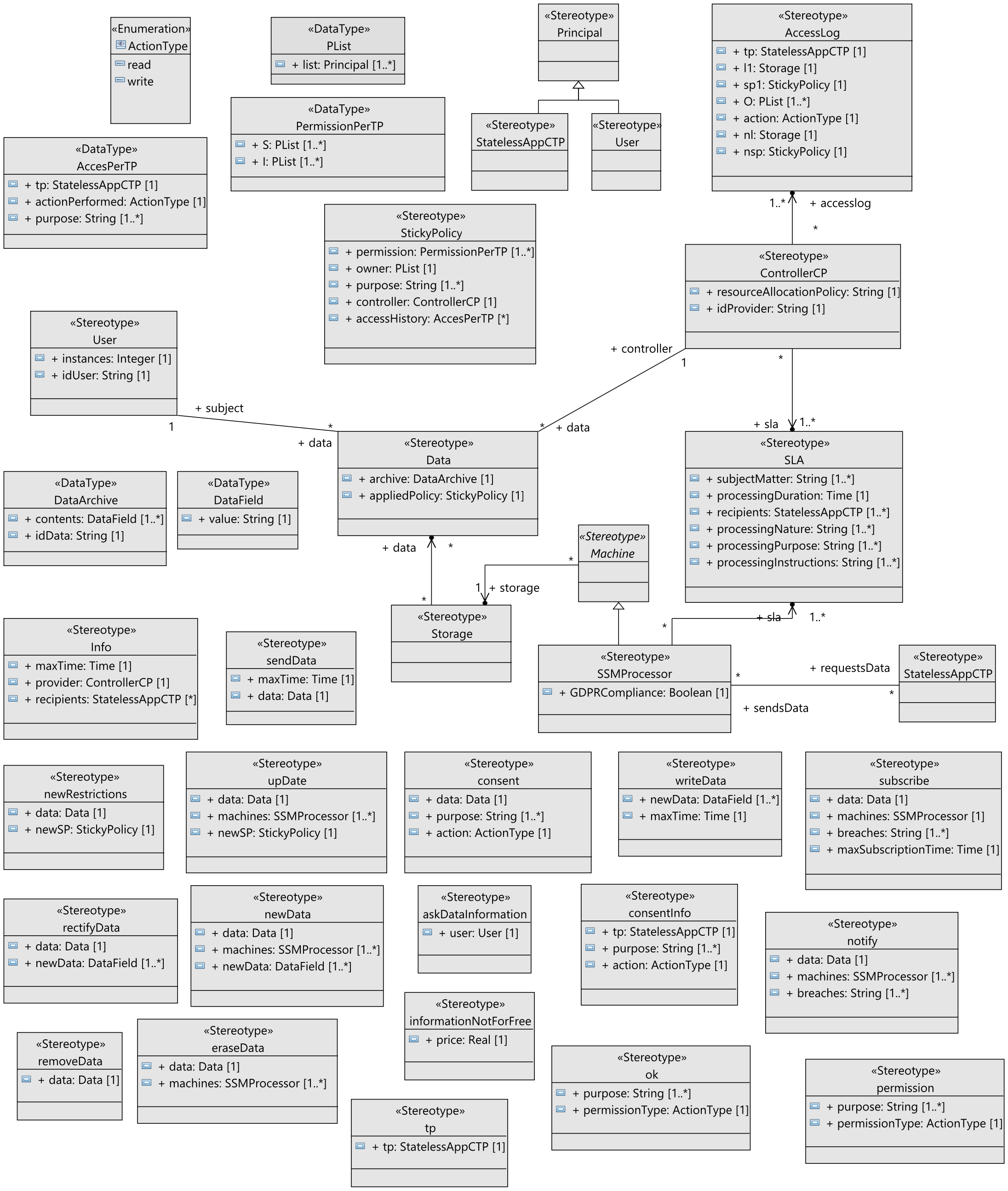

Figure A.2. Model4_DataCTrack profile: Associations and properties of cloud-GDPR interaction stereotypes.

the list of third parties who are allowed to access the data so far. The nature of the treatment and the purpose are the following two attributes, where the latter must match the one indicated in the SP defined by the user and are defined as *string* arrays. Finally, the attribute *processingInstructions* models the set of directions given by the controller to regulate data processing.

The *ControllerCP* stereotype includes two attributes: *resourceAllocationPolicy* and *idProvider*. The first models the type of policy that the controller uses to allocate its resources. The second attribute, defined as a *string* type, models the information about the controller it must include in each contract as *spContact*, which is the cloud service provider. The remaining attributes result from the use of end classifiers in the associations of this stereotype. As stated above, these are represented by an arrow with a dot at one end of an association and indicate that the marked stereotype will be an attribute of the

stereotype at the other end. It is also worth noting that the multiplicity of the end with the dot becomes that of the resulting attribute. Thus, having a multiplicity of one-or-many in the marked stereotype implies that the resulting attribute represents a set of elements of that type. Therefore, *ControllerCP* receives two attributes named *accessLog* and *sla* of *AccessLog* and *SLA* types, respectively.

In contrast, the few primitive type attributes in this diagram are mostly *strings*, as represented by the *ControllerCP* or *SLA* stereotypes.

The *Data* stereotype represents the data that belongs to a certain user or set of users (only in the case of combined data). For this stereotype, it is necessary to include two specific data types, namely *DataArchive* and *DataField*. *DataArchive* models the structure of a data file, being composed of an identifier, *idData*, and its contents, *contents*. The content of an archive consists of a group of fields (*DataField* type), and each one, in turn, contains a value, which is an attribute of *string* type. In addition, the *Data* stereotype includes the sticky policy that is applied to it (*appliedPolicy* attribute). The *Storage* attribute, in turn, is an attribute of *Machine*, which is abstract, so it will be inherited by the *SSMProcessor* and *StatelessComputationMachine* stereotypes. The processors represent the machines that store and maintain the data at all times, although the computing machines will only occasionally store data (provided by a *SSMProcessor*) when processing it via the *StatelessAppCTP* that requested such data.

B OCL RULES

OCL rules	
**Name**	**no_empty_racks**
Severity	ERROR
Context	Rack
Description	This rule validates that attributes machinesPerBoard and boards in stereotype Rack (self.machinesPerBoard and self.boards) are both greater than 0 with a logical AND operation.
Specification	`self.machinesPerBoard>0 and self.boards>0`
**Name**	**cpu_cores_and_flops_greater_than_0**
Severity	ERROR
Context	CPU
Description	Similarly to the previous rule this one checks that the number of cores and FLOPs of a CPU are both greater than 0.
Specification	`self.cores>0\ and\ self.FLOPs>0`
**Name**	**latency_name_not_empty**
Severity	ERROR
Context	Latency
Description	Validates that the latency's name is not an empty string by checking its size (number of characters) is greater than zero
Specification	`self.name.size()>0`
**Name**	**size_value_greater_than_0**
Severity	ERROR
Context	Size
Description	Assures that the value for any attribute of type Size is greater than 0
Specification	`self.value>0`
**Name**	**time_value_greater_than_0**
Severity	ERROR
Context	Time
Description	Checks that the value of any attribute of type Time (self.value) is greater than 0
Specification	`self.value>0`
**Name**	**bandwidth_value_greater_than_0**
Severity	ERROR
Context	Bandwidth
Description	Checks that the value of any attribute of type bandwidth (self.value) is greater than 0
Specification	`self.value>0`

OCL rules	
**Name**	**numberOfDrivers_greater_than_ns**
Severity	Error
Context	Storage
Description	Validates that the value of attribute numberOfDrivers of type Storage (self.numberOfDrivers) is greater than 0
Specification	`self.numberOfDrivers>0`
**Name**	**sendData_maxTine_value_greater_than_0**
Severity	ERROR
Context	sendData
Description	This rule checks that the time value for the attribute maxTime of the sendData message is greater than 0
Specification	`self.maxTime.value>0`
**Name**	**paste_maxTine_value_greater_than_0**
Severity	ERROR
Context	pasteData
Description	This rule assures that the value of the maxTime attribute of pasteData stereotypes is a number greater than zero
Specification	`self.maxTime.value>0`
**Name**	**combine_maxTine_value_greater_than_0**
Severity	ERROR
Context	combineData
Description	This rule checks that the time value for the attribute maxTime of the combineData message is greater than 0
Specification	`self.maxTime.value>0`
**Name**	**maxSubTime_greater_than_0**
Severity	ERROR
Context	Subscribe
Description	This rule checks that the attribute maxSubscriptionTime in Subscribe type is greater than zero
Specification	`self.maxSubscriptionTime.value>0`
**Name**	**machine_contains_data_to_rectify**
Severity	ERROR
Context	newData
Description	Validates that the set of data to rectify with the contents on the message newData is located in all of the machines which the message is destined to. This is achieved by verifying that, for all the machines in the list of the newData message (self.machines), the data included in the message (self.data) is included in every list of data inside the machine (m.data)
Specification	`self.machines-->forAll(m | m.data-->includes(self.data))`
**Name**	**machine_contains_data_to_erase**
Severity	ERROR
Context	eraseData
Description	Similarly to the previous rule, this one checks that the set of data to erase on the message eraseData is located in all of the destination machines of the message.
Specification	`self.machines-->forAll(m | m.data-->includes(self.data))`
**Name**	**machine_contains_data_to_subscribe_to**
Severity	ERROR
Context	subscribe
Description	Alike the former two rules, this one checks that the set of data which the controller wants to subscribe to is present in all of the destination machines of the message.
Specification	`self.machines-->forAll(m | m.data-->includes(self.data))`

<table>
<tr><th colspan="2">OCL rules</th></tr>
<tr><td>Name
Severity
Context
Description

Specification</td><td>location1_machine_not_under_sla_with_controller
ERROR
ControllerCP
This rule checks that the processor contained in accesslog from which data has been obtained for the operation is under SLA with the controller of said data. To do this it accesses the list of accesslogs of the controller (self.accesslog) and checks, for all of them, that it exists at least one SLA in the controller list which is included in the SLA list of the location1 machine of the log (log.location1.sla)
<pre>
self.accesslog-->
  forAll(log | self.sla -->
               exists(sla | log.location1.sla-->includes(sla)))
</pre></td></tr>
<tr><td>Name
Severity
Context
Description

Specification</td><td>sourceMachine_not_under_sla_with_controller
ERROR
ControllerCP
This rule validates that the machine containing the source copy of data is under SLA with the controller. First, it gets the list of SLAs for the controller included inside the sticky policy of the log of the controller (self.accesslog.sp.controller.sla), then it checks that it exists (exists operation) at least one sla in said list which is included (includes operation) in the list of SLAs in the source machine contained in the same sticky policy of the log (self.accesslog.sp.sourceMachine.sla)
<pre>
self.accesslog.sp.controller.sla -->
    exists(sla | self.accesslog.sp.sourceMachine.sla-->
                 includes(sla))
</pre></td></tr>
<tr><td>Name
Severity
Context
Description

Specification</td><td>duplicatesMachine_not_under_sla_with_controller
ERROR
ControllerCP
This rule validates that the machine containing the source copy of data is under SLA with the controller. First it gets the list of SLAs for the
<pre>
self.accesslog.sp.duplicates ->
   forAll(m | self.accesslog.sp.controller.sla ->
              exists(sla | m.sla->includes(sla)))
</pre></td></tr>
<tr><td>Name
Severity
Context
Description

Specification</td><td>cpu_cores_and_flops_greater_than_0
ERROR
CPU
Similarly to the previous rule this one checks that the number of cores and FLOPs of a CPU are both greater than 0.
<code>self.cores>0 and self.FLOPs>0</code></td></tr>
<tr><td>Name
Severity
Context
Description

Specification</td><td>latency_name_not_empty
ERROR
Latency
Validates that the latency's name is not an empty string by checking its size (number of characters) is greater than zero
<code>self.name.size()>0</code></td></tr>
<tr><td>Name
Severity
Context
Description
Specification</td><td>size_value_greater_than_0
ERROR
Size
Assures that the value for any attribute of type Size is greater than 0
<code>self.value>0</code></td></tr>
</table>

OCL rules	
**Name**	**accessHistory_tp_not_in_recipients_list**
Severity	ERROR
Context	ControllerCP
Description	In this rule the list of third parties who accessed the data is first accessed, this is done through the sticky policy attribute (sp) of the controller's accesslog (self.accesslog.sp.accessHistory). Then, it is check for all them (forAll operation) that for all the users (second forAll operation) in the list of owners (self.accesslog.sp.owners) the list of recipients of their user contract (ow.bindingContract.recipients) includes the third party in the accessHistory attribute (his.tp). In this way it is ensured that data is not accessed by any tp that the users have not been informed of. Note that this could have been done with StickyPolicy as starting point, but with the additional navigation the error is thrown by the controller which is the entity that would manage this situation in a real scenario.
Specification	`self.accesslog.sp.accessHistory-->`<br>`forAll(his | self.accesslog.sp.owners-->`<br>`forAll(ow | ow.bindingContract.recipients-->`<br>`includes(his.tp)))`
**Name**	**no_empty_newData_fields**
Severity	ERROR
Context	newData
Description	This rule is meant to ensure that the data introduced in the newData messages does not infringe the data accuracy RGPD principle by introducing empty fields. To do this, it is checked that for all the fields in the newData attribute of the message (self.newData), the size (number of characters of the string) is greater than 0
Specification	`self.newData-->forAll(f | f.value.size()>0)`
**Name**	**no_empty_write_fields**
Severity	ERROR
Context	rectifyData
Description	Similarly to the previous rule, this one validates that no empty fields are introduced in the write message
Specification	`self.newContent-->forAll(f | f.value.size()>0)`
**Name**	**sendData_timeunit_not_hours_or_minutes**
Severity	WARNING
Context	combineData
Description	Notes that the units of time for the maximum storage time of data are smaller than usual. The way this is check is the exact same as in the previous rule
Specification	`self.maxTime.unit=TimeUnit::h or self.maxTime.unit=TimeUnit::min`
**Name**	**newData_destinatnion_machines_comply_with_GDPR**
Severity	ERROR
Context	newData
Description	This rule ensures that all of the machines included as destinations of a newData message are marked as compliant with the GDPR, just like rule 10 does for upDate.
Specification	`self.machines-->forAll(m | m.GDPRCompliance=true)`
**Name**	**eraseData_destinatnion_machines_comply_with_GDPR**
Severity	ERROR
Context	eraseData
Description	This rule checks that the destination machines of an eraseData message comply with the GDPR in the same way that the previous rules.
Specification	`self.machines-->forAll(m | m.GDPRCompliance=true)`
**Name**	**subscribe_destinatnion_machines_comply_with_GDPR**
Severity	ERROR
Context	subscribe
Description	This rule ensures that all of the machines included as destinations of a subscribe message are marked as compliant with the GDPR.
Specification	`self.machines-->forAll(m | m.GDPRCompliance=true)`

OCL rules	
**Name**	**notify_destinatnion_machines_comply_with_GDPR**
Severity	ERROR
Context	notify
Description	in the same way that the previous rules do it, this rule checks that the destination machines of a notify message comply with the GDPR.
Specification	`self.machines-->forAll(m | m.GDPRCompliance=true)`
**Name**	**pasteData_machine2_complies_with_GDPR**
Severity	ERROR
Context	pasteData
Description	This rule checks that the machine2 of the pasteData message, in which data is going to be copied, complies with the GDPR standards.
Specification	`self.machine2.GDPRCompliance=true`
**Name**	**combineData_machine2_complies_with_GDPR**
Severity	ERROR
Context	combineData
Description	This rule checks that the machine2 of a combineData message, in which the data set resulting of a combine operation is going to be stored, complies with the GDPR standards.
Specification	`self.machine2.GDPRCompliance=true`
**Name**	**consent_machine_complies_with_GDPR**
Severity	ERROR
Context	consent
Description	This rule checks that the machine of a consent message, which will be accessed by a third party if consent is given, complies with the GDPR standards.
Specification	`self.machine.GDPRCompliance=true`
